# Retarding the capacity fading and voltage decay of Li-rich Mn-based cathode materials *via* a compatible layer coating for high-performance lithium-ion batteries†

**DOI:** 10.1039/d4ra03660c

**Published:** 2024-08-19

**Authors:** Shaofeng Liu, Haifeng Yue, Yan Mo, Liang Luo, Xiaozhen Wu, Shunyi Yang, Youyuan Huang, Guohui Yuan

**Affiliations:** a School of Chemistry and Chemical Engineering, Harbin Institute of Technology Harbin 150001 People's Republic of China ygh@hit.edu.cn; b Shenzhen BTR Nanotechnology Co., Ltd Shenzhen 518106 People's Republic of China huangyouyuan@btrchina.com

## Abstract

Li-rich Mn-based layered oxides have been considered as the most promising cathode candidate for high energy density lithium ion batteries. However, the practical application of Li-rich Mn-based layered oxides is hindered due to the capacity fading and voltage decay accompanied with structure transition from the layered structure to spinel phase during cycling. Herein, a facile surface structure repair *via* Ce modification is reported. The structural analysis of the bulk and coating layer was carried out using XRD, XPS, SEM and TEM, which confirmed the successful doping of Ce and formation of a Li_2_CeO_3_ coating on the surface. The modified sample LLO-2 delivers a discharge specific capacity of 263.5 mA h g^−1^ at 0.1C and capacity retention rate with 88.1% at 0.2C after 100 cycles compared to 250.2 mA h g^−1^ and 75.6% for the pristine sample. The enhanced performance could be because Ce doping enlarges the lattice parameter, which may contribute to accelerating the Li^+^ diffusion rate. Moreover, the newly formed Li_2_CeO_3_ coating with oxygen vacancies could inhibit the loss of lattice oxygen and protect the electrode surface by suppressing the attack from the electrolyte. This work provides an effective approach to design Li-rich Mn-based layered oxides with improved electrochemical performance.

## Abstract 2

Ce doping and Li_2_CeO_3_ coating are favorable for enhancing the electrochemical performance of Li-rich Mn-based layered oxides.

## Graphical abstract

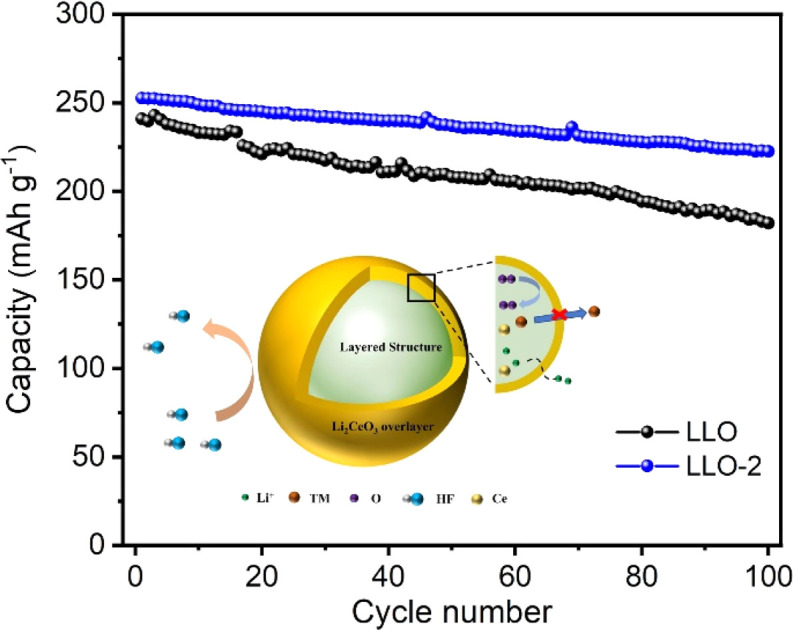

## Introduction

With the rapid development of portable electronics, electric vehicles (EVs) and hybrid electric vehicles, lithium-ion batteries (LIBs) with higher energy density, low cost and long cycle life have attracted extensive attention.^1–5^ Among present lithium-ion cathode materials, lithium-rich manganese-based layered oxides (LLOs), expressed as *x*Li_2_MnO_3_·(1 − *x*)LiMO_2_ (M = Ni, Co and Mn), are regarded as a suitable candidate for next-generation cathodes for EV-LIBs owing to their high theoretical capacity over 250 mA h g^−1^, which originates from the cationic and anionic redox reaction during the electrode process.^6–8^ Among various Li-rich materials, Li_1.2_Ni_0.2_Mn_0.6_O_2_ not only has lower cost but also is more environmentally friendly than other Co-containing counterparts, which is much more suitable for large-scale energy storage and EV batteries.^9^ However, this material suffers from low initial coulombic efficiency, severe capacity fading and continuous potential dropping due to the irreversibility of oxygen release, structure degradation and transition metal dissolution, which cannot satisfy the requirements for commercialized LIBs.^10,11^

To overcome the above-mentioned problems and enhance the cycling performance of LLOs, numerous modification strategies including bulk doping, surface coating, and acid pretreatment have been investigated to reinforce the structure of LLOs.^12,13^ For example, a series of elements have been introduced into the internal structure of LLOs to enhance the structural integrity and alleviate the oxygen release, including Al, Mg, K, F, Na and dual ions.^14–19^ However, lattice mismatch between the active host and doping guest leads to the deformation of the structure during long-term cycling; therefore, atomic-scale doping is still challenging. On the other hand, surface coatings such as Al_2_O_3_, TiO_2_, SnO_2_, AlF_3_, Li_2_SiO_3_, Li_3_PO_4_, Li_2_ZrO_3_, LiTaO_3_, and LiVO_3_ have been reported to protect the active materials, which can alleviate severe side reactions with the electrolyte due to the formation of a barrier between the active material and the electrolyte.^20–28^ Because of the poor electronic and ionic transport of LLOs, coating Li-containing oxides are indispensable, which are believed to enhance Li^+^ conduction at the interface. Sun *et al.* demonstrated that better electrochemical properties could be obtained by coating LiAlO_2_ because of its good conductivity for Li ions.^29^ Yang *et al.* discovered that the spinel phase-induced by YF_3_ with a 3D Li^+^ diffusion channel can improve the rate performance, and F doping could inhibit the phase transformation, which make it a prospective candidate for layered oxides surface coating.^30^ Moreover, Kang *et al.* reported that surface LiCeO_2_ coating delivers a better capacity retention and less oxygen release owing to the abundant oxygen vacancies of LiCeO_2_.^31^

In this study, inspired by the superiority of surface coating and bulk doping, we synthesized Ce-modified LLOs *via* the high-temperature solid-phase method. The morphology and structure were systematically analyzed using scanning electron microscopy (SEM), transmission electron microscopy (TEM), X-ray powder diffraction (XRD), and X-ray photoelectron spectroscopy (XPS). Specifically, the designed material LLO-2 delivers a high discharge 263.5 mA h g^−1^ at 0.1C with a coulombic efficiency of 88.1% and excellent capacity retention of 88.1% after 100 cycles compared with the pristine one. The above enhancement is ascribed to the formation of the Li_2_CeO_3_ coating, which can inhibit the attack from the electrolyte, suppress the side reaction, and reduce the oxygen release in the bulk. Moreover, the doping of Ce and the Li_2_CeO_3_ coating also improve the lithium-ion conductivity. Meanwhile, the oxygen loss during cycling is substantially retarded because of the formed Li_2_CeO_3_ containing abundant oxygen vacancy. This work provides an effective method to solve the poor electrochemical performance of lithium-rich materials.

## Experimental

### Material preparation

The precursor Ni_0.25_Mn_0.75_(OH)_2_ was synthesized with a co-precipitation method. Specifically, NiSO_4_·6H_2_O solution and MnSO_4_·H_2_O solution with a molecular ratio of 1 : 3 (Ni : Mn) were pumped into a 50 L batch reactor containing NaOH and NH_3_·H_2_O. The pH was adjusted by adding NH_3_·H_2_O, and the temperature was maintained at 50 °C. Nitrogen gas was continuously fed into a reactor to prevent the oxidation of metal ions. Finally, the obtained precipitate was washed and filtered with deionized water and dried in a vacuum oven at 120 °C for 24 h to attain hydroxide precursor.

To prepare Ce-modified Li_1.2_Ni_0.2_Mn_0.6_O_2_, Ce(OH)_4_ was added to the stoichiometric precursor and Li_2_CO_3_ (in excess of 5%), and the above mixture was placed in a silicon nitride grinding vial with silicon nitride balls. The ball milling was performed using a SPEX 8000D mill for 20 min, and the process was repeated 3 times. The received mixture was annealed at 500 °C for 5 h and 900 °C for 12 h in a box furnace in air with a heating rate of 5 °C min^−1^. The amounts of Ce were set at 3 wt%, 5 wt% and 7 wt% by changing the adding weight of Ce(OH)_4_, which were denoted as LLO-1, LLO-2, and LLO-3, respectively. For comparison, pristine Li_1.2_Ni_0.2_Mn_0.6_O_2_ was synthesized with the same process without adding Ce(OH)_4_ and was denoted as LLO. Furthermore, 5 g of LLO and 0.25 g of Ce(OH)_4_ were dispersed in 20 mL ethanol for 6 h of ultrasonication and then the solution was transferred in an oven at 80 °C for 6 h. Finally, the received mixture was annealed at 600 °C for 12 h in a box furnace in air with a heating rate of 5 °C min^−1^. The final product was denoted as LLO-4.

### Material characterization

The chemical compositions of the prepared samples were accurately analyzed by inductively coupled plasma optical emission spectroscopy (ICP-OES, Optima 7300 DV, PerkinElmer Co., USA). Powder X-ray diffraction (Bruker AXS D8, Bruker AXS GmbH, Germany) was applied to characterize the crystal structure of the synthesized materials with Cu Kα radiation at a low scan rate of 0.5° min^−1^ between 10 and 75°. The XRD patterns were refined by the Rietveld method using the Fullprof software. Thermogravimetric analysis (TGA) was carried out on a TG instrument (Q50 TGA) at a heating rate of 5 °C min^−1^ in air flow from room temperature to 950 °C. SEM (HITACHI, S-4800) and TEM (FEI Tecnai G2 F20) were used for morphological and structure assessment. Focused ion beam-(FIB) SEM (FIB-SEM) (FEI Helios Nano Lab 660) was carried out to observe the cross-sectional morphology of the materials. XPS was performed to analyze the material's elemental composition and electronic state on a Thermo Fisher Scientific 250Xi (USA) with an Al anode source operating at 1486.6 eV. The C 1s peak was calibrated to 284.8 eV for each data set before comparison.

### Electrochemical measurements

A two-electrode CR2016 coin-type half-cell was prepared to test the electrochemical performance of the cathode materials, which was assembled in a glovebox. The cathode electrode slurry was prepared by mixing 80 wt% active material, 10 wt% carbon black (Super P) and 10 wt% PVDF (polyvinylidene fluoride) with *N*-methyl-2-pyrrolidone. The slurry was coated onto a thin Al foil and vacuum-dried at 120 °C for 12 h. The dried electrode was pressed and punched into circular films with a loading mass of 2.0 mg cm^−2^. The half-cell was prepared using lithium foil as the anode. Also, a porous polypropylene-based membrane (Celgard-2400) was used to separate the positive and negative electrodes. The electrolyte was obtained by dissolving 1 M LiPF_6_ in a solvent mixture of EMC, DMC and EC (1 : 1 : 1 in volume ratio). Finally, the coin cells were tested on a battery system at different current densities in the range of 2.0–4.8 V (*vs.* Li^+^/Li). Electrochemical impedance spectroscopy (EIS) study was conducted in the frequency range from 100 kHz to 0.01 Hz at an AC voltage amplitude of 5 mV. Cyclic voltammetry (CV) tests were performed using a PARSTAT (VMC) at a scanning rate of 0.1 mV s^−1^ in the range of 2.5–4.8 V (*vs.* Li^+^/Li).

## Results and discussion

**Fig. 1:**
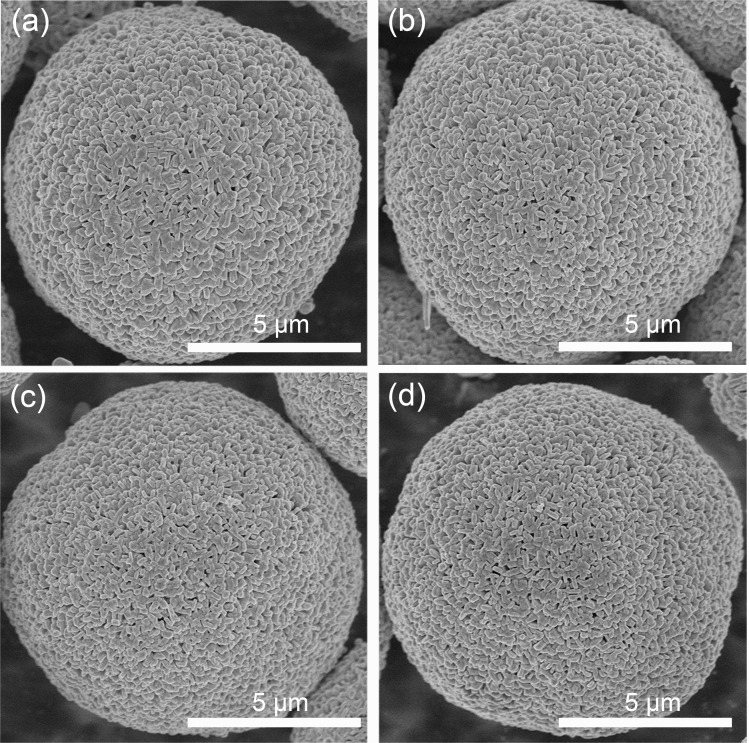
SEM images of (a) LLO, (b) LLO-1, (c) LLO-2 and (d) LLO-3.

As shown in Fig. S1,† the as-prepared precursor exhibits a well-defined sphere morphology with a thin primary particle. Fig. 1 shows the SEM images of the pristine and Ce-modified samples, which display a typical spherical morphology with a mean size of about 9 μm. LLO, LLO-1 and LLO-2 were composed with analogous micron-sized primary particles, which prove that the introduction of Ce will not change the morphology of the pristine (Fig. 1a–c and S2a–c†). The length of the primary particle size was 200 ± 45.6 nm for the pristine and Ce-modified samples. However, the surface of primary grains becomes rough slightly for LLO-3, which suggests that too much addition of Ce may affect the growth of pristine grains (Fig. 1d and S2d†). Thermogravimetric analysis (TGA) was performed to investigate to the formation process of LLO and mass loading of Ce in LLO-2. As shown in Fig. S3,† there is no obvious mass change before 200 °C. The weight loss between 200 and 500 °C is considered to be due to the decomposition of the precursor, Li_2_CO_3_ and Ce(OH)_4_, which is accompanied by the formation of Li-rich Mn-based layered oxides. The higher 1.28% weight loss of LLO-2 should be due to the decomposition of Ce(OH)_4_, which is equal to 4.98% mass loading of Ce. The region between 500 and 800 °C is generally considered as the evolution of oxygen and Li. Meanwhile, we also compared the ICP-OES results of LLO with LLO-2 to verify the atom contents. It is clear that the Ce loading of LLO-2 determined by ICP-OES is comparable to the TGA results (Table S1†).

**Fig. 2:**
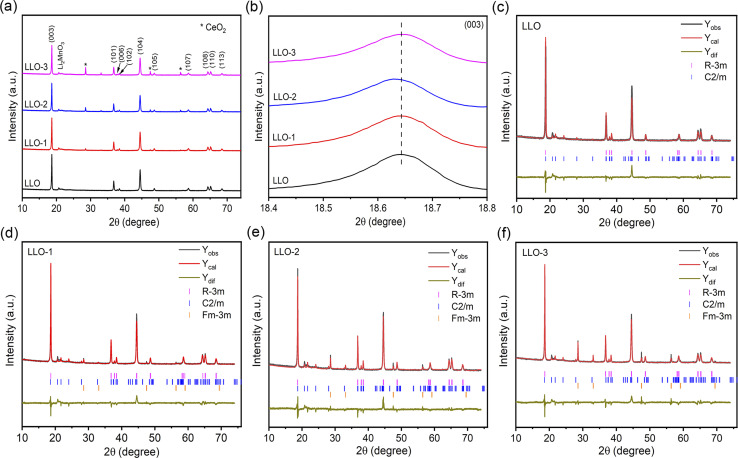
(a) XRD patterns of all the samples, (b) magnified imaged of the (003) peak, Riveted refinement results of (c) LLO, (d) LLO-1, (e) LLO-2 and (f) LLO-3.

XRD was performed to study the crystal structure and phase composition of electrode materials. As illustrated in Fig. 2a, all the samples present a typical hexagonal α-NaFeO_2_ structure with a space group of *R*3̄*m*, which points that the addition of Ce will not affect the crystalline of LLOs.^32^ Besides, the weak peaks in the range of 20–23° correspond to monoclinic Li_2_MnO_3_ phase with a *C*2/*m* space group, which is due to the superlattice ordering of Li and Mn in the transition metals layer.^8,33^ Also, the obvious splitting peaks of (006)/(102) and (108)/(110) demonstrate that the layered structure of all the samples are well-maintained.^34,35^ As shown in Fig. 2b, the enlarged (003) peaks of LLO-1 and LLO-2 shift to left slightly compared with that of LLO, which may be ascribed to the successful introduction of Ce into the bulk.^36^ To further identify the effect of Ce doping on the crystalline structure, the Rietveld refinement of XRD patterns was conducted (Fig. 2c–f), and the related Rietveld refinement parameters are presented in Table S2.† As listed in Table S2,† the cell parameters *c* of LLO-1 and LLO-2 exhibit a slight enlargement with the increase in Ce addition, suggesting that Ce has been successfully introduced into the bulk phase. However, the cell parameter *c* of LLO-3 decreases slightly, which may be due to the excessive addition of Ce, resulting in an uneven coating. Moreover, the peak intensity of *I*(003)/*I*(104) of LLO, LLO-1, LLO-2 and LLO-3 are 1.698, 1.702, 1.736 and 1.476, respectively, which reveals that LLO-2 has the lowest degree of Li–Ni anti-sites, which may contribute to the improvement of the rate capability. The LLO-3 displays the lowest *I*(003)/*I*(104) value, which is due to the formation of the cubic CeO_2_ phase with a *Fm*3̄*m* space group.

**Fig. 3:**
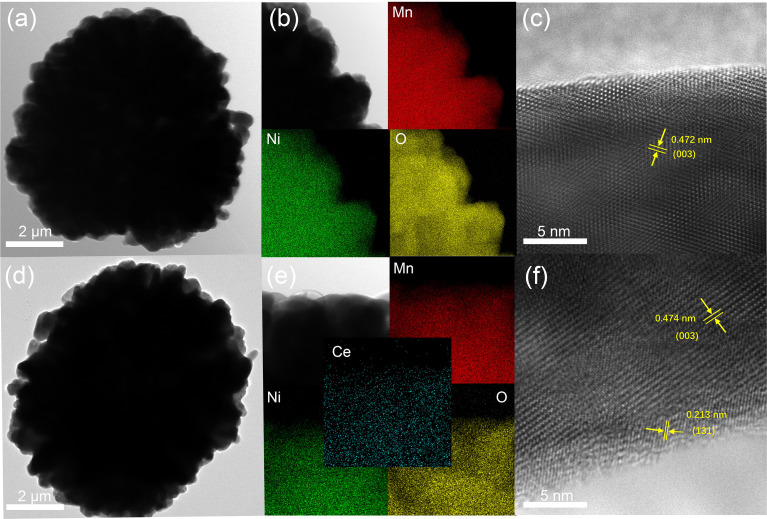
(a) TEM image, (b) EDS elemental mapping and (c) HRTEM image of LLO, (d) TEM image, (e) EDS elemental mapping and (f) HRTEM image of LLO-2.

In addition, to further identify the structure and elemental distribution of the samples, TEM and energy dispersive X-ray spectroscopy (EDS) mapping were carried out. Fig. 3a shows the TEM of LLO with a particle size of about 9 μm, and the distribution of Ni, Mn, O elements is uniform, in line with the expectation (Fig. 3b). The high-resolution transmission electron microscopy (HRTEM) of LLO exhibits a clear lattice fringe from the bulk to the surface region with an interplanar spacing of 0.472 nm, which is well in good accordance with the (003) plane of the layered structure (Fig. 3c).^37^Fig. 3d and e exhibit the TEM and EDS elemental mapping of LLO-2, demonstrating the homogeneous distribution of Ce element. Interestingly, HRTEM shows that LLO-2 is covered by a uniform coating with a thickness of about 2–3 nm while the internal structure remains as that of the pristine one (Fig. 3f). Also, the new interplanar spacing of the lattice fringes at the edge region was measured to be 0.213 nm, which is assigned to the (131) crystal plane of Li_2_CeO_3_.^38^ However, the characteristic peaks of Li_2_CeO_3_ are not observed in the XRD patterns, which may be because the amount of Li_2_CeO_3_ is below the detection limit of the XRD equipment, consistent with the previous reports.^31,38^ The existence of Li_2_CeO_3_ may exhibit an obvious enhancement in the rate performance of the cathode due to its high lithium ion conductivity.^39^ Also, the above unpredicted phase may enhance the structural integrity of the bulk, which may prevent the damage from the electrolyte.^40^ In addition, the TEM image and EDS mapping of LLO-1 and LLO-3 are exhibited in Fig. S4,† in which the distribution of Ce disperses unevenly in LLO-3 because of the excessive addition of Ce.

**Fig. 4:**
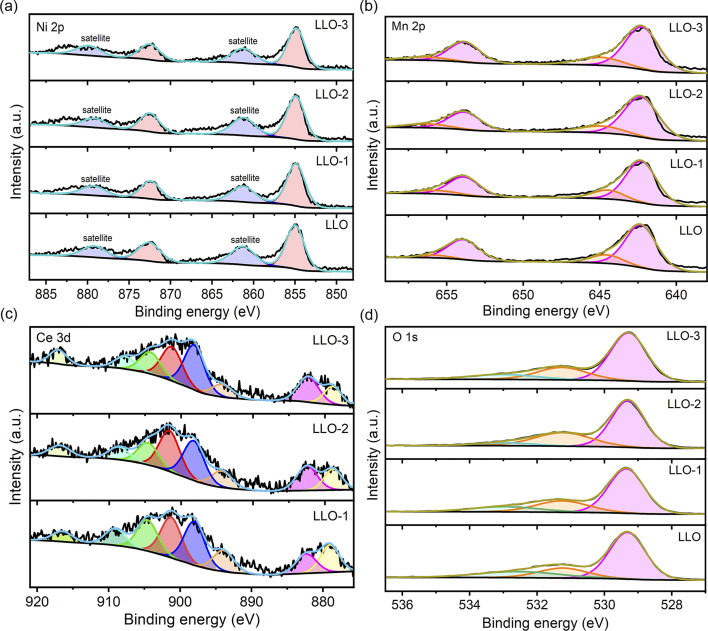
XPS spectra of LLO, LLO-1, LLO-2 and LLO-3 for (a) Ni 2p, (b) Mn 2p, (c) Ce 3d and (d) O 1s.

To further identify the surface composition and chemical state of the prepared samples, XPS was performed, and the corresponding XPS survey spectra are presented in Fig. S5.† The high-resolution XPS spectra of Ni 2p, Mn 2p, Ce 3d and O 1s of all the samples are presented in Fig. 4. It was found that the XPS characteristic peaks of Ni 2p and Mn 2p of the modified samples show no shift compared with that of pristine LLO. The binding energy of Ni and Mn is 855.1 eV and 642.3 eV, respectively, representing Ni^2+^ and Mn^4+^ in both pristine and Ce-modified materials, which are in good accordance with recent reports (Fig. 4a and b).^37^ As shown in Fig. 4c, three characteristic peaks of Ce 3d are observed at 916.9 and 897.9 eV, 908.5 and 893.9 eV, and 901.4 and 882.4 eV, corresponding to Ce^4+^ except for LLO, which confirms the presence of CeO_2_ and Li_2_CeO_3_. Moreover, the fitting analysis of O 1s spectra reveals three distinct characteristic peaks at 529.3 eV, 531.3 eV and 532.9 eV, which correspond to the lattice oxygen, oxygen vacancy and chemisorbed oxygen, respectively (Fig. 4d).^31^ From the XPS fitting results, there are much more oxygen vacancies on the Ce-modified materials than that on the pristine one, and LLO-2 contains the highest amount of oxygen vacancies. Also, the proportion of oxygen vacancies in LLO and LLO-2 was calculated to be 18.3% and 29.4%, respectively. The oxygen vacancy content of LLO-3 is lower than that of LLO-2, which may be due to the much more Ce doping into the lattice. To the best of our knowledge, oxygen vacancy is regarded as a critical factor to enhance the reversibility of anionic redox and suppress oxygen release.^41^ Therefore, much more existence of oxygen vacancy in LLO-2 may effectively stabilize the lattice oxygen and enhance the cycle stability of the electrode.

**Fig. 5:**
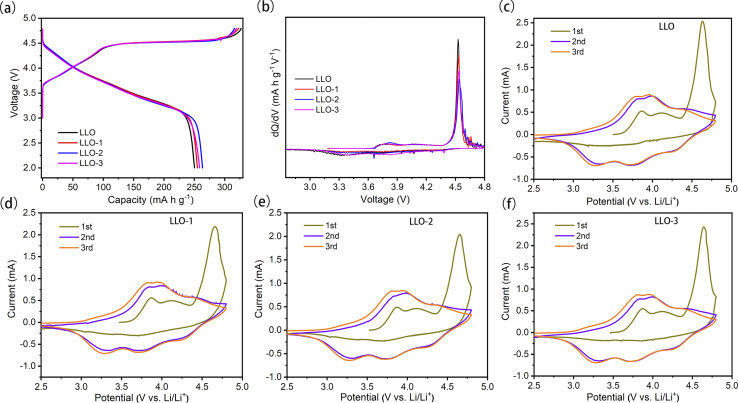
(a) Initial charge–discharge curve profiles of LLO, LLO-1, LLO-2 and LLO-3 at 2.0–4.8 V and 0.1C and (b) corresponding d*Q*/d*V* curves, CV curves at 0.1 mV s^−1^ between 2.5 and 4.8 V of (c) LLO, (d) LLO-1, (e) LLO-2, (f) LLO-3.

To evaluate the effect of Ce modification on the electrochemical performance of the prepared samples, the initial charge–discharge curves tested at 0.1C in the voltage range of 2.0–4.8 V are depicted in Fig. 5a. As can be seen from the curves, all the samples exhibit similar charge–discharge curves. The smooth slope below 4.45 V corresponds to the extraction of Li^+^ and oxidation of Ni^2+^. Also, the long plateau region at about 4.5 V can be attributed to the simultaneous removal of lithium and lattice oxygen from Li_2_MnO_3_.^17,42^ The initial discharge-specific capacities of pristine LLO, LLO-1, LLO-2 and LLO-3 are 250.2, 255.1, 263.5 and 258.6 mA h g^−1^, and the corresponding initial coulombic efficiencies (ICE) are 75.5%, 85.3%, 88.1% and 87.2%, respectively. It is obvious that the ICE and discharge capacity increase partially with the increase in the Ce addition amount. The enhancement of ICE may be ascribed to the suppression of lattice oxygen evolution, which is in accordance with the d*Q*/d*V* results (Fig. 5b). Also, the formed Li_2_CeO_3_ promotes the kinetic diffusion of Li^+^, which may contribute to the enhancement of the capacity. On the other hand, the slight decrease in the discharge specific capacity of LLO-3 compared with LLO-2 may be due to the superabundant coating formed, which is detrimental to the de-intercalation of Li^+^.

Fig. 5b shows the corresponding d*Q*/d*V* profiles; the oxidation peak at 4.5 V is attributed to the activation of Li_2_MnO_3_. The weaker anode oxidation peaks of the modified samples demonstrate that the continuous oxygen loss is effectively inhibited. This is due to the newly formed Li_2_CeO_3_ on the surface of the modified samples, which is rich in oxygen vacancies.^38^ The newly formed oxygen vacancy could act as the active site for the spillover of O^2−^, therefore alleviating the massive loss of lattice oxygen and enhancing the reversibility of the anion-redox reaction, which is in line with the previous results.^31,36,43–45^ To further figure out the electrochemical behavior of the samples, cyclic voltammetry (CV) tests were conducted at 0.1 mV s^−1^ for the initial three cycles, and the corresponding curves are plotted in Fig. 5c–f. All the samples exhibit three oxidation peaks in the first cycle. The oxidation peaks at about 4.0 V resulted from the oxidation of Ni^2+^ to Ni^4+^, which is accompanied by the de-intercalation of Li^+^ from the layered phase.^46^ Moreover, the oxidation peaks at 4.6–4.7 V in the CV curves are attributed to the irreversible activation process of Li_2_MnO_3_ along with the evolution of lattice oxygen. The newly formed oxidation peaks at 3.6–4.4 V in the second and third cycles are due to the oxidation of Ni^2+^ to Ni^4+^. The reduction peaks at about 3.7 V and 3.2 V mainly originate from the reduction of Ni^4+^ to Ni^2+^ and Mn^4+^ to Mn^3+^ in the second and third cycle, respectively.^41^ The oxidation peak at 4.5 V of LLO-2 is weaker than that of LLO, which proves that the activation of Li_2_MnO_3_ is suppressed significantly, in accordance with the d*Q*/d*V* results.

**Fig. 6:**
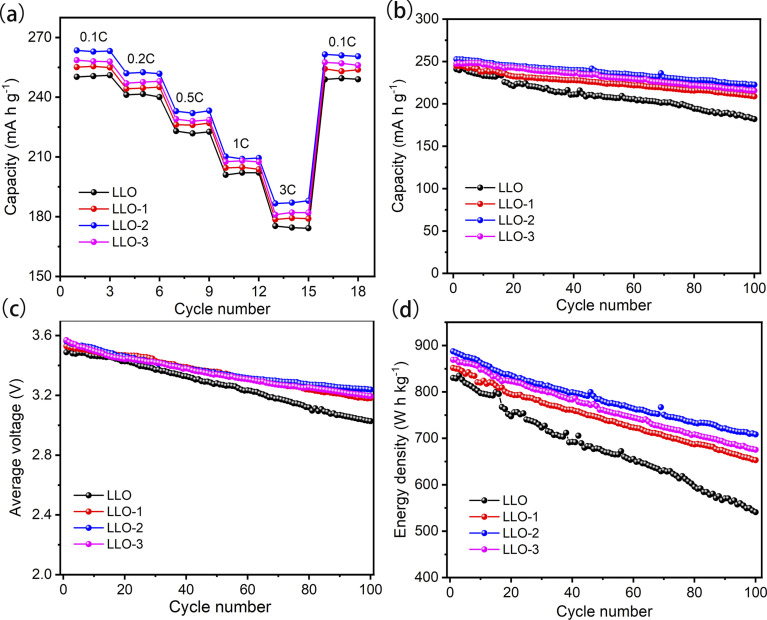
(a) Rate performance, (b) capacity retention, (c) median voltage decay, (d) energy density of all the samples at 0.2C between 2.0 and 4.8 V.

The rate capabilities of pristine and Ce-coating samples are displayed in Fig. 6a. The electrodes are charged and discharged at a current rate of 0.1C, 0.2C, 0.5C, 1C, and 3C for 3 cycles between 2.0 V and 4.8 V. As can be seen from Fig. 6a, the modified samples exhibit better rate performance than the pristine one. LLO-2 shows discharge capacities of 263.5 mA h g^−1^, 252.0 mA h g^−1^, 233.2 mA h g^−1^, 210.2 mA h g^−1^, 186.6 mA h g^−1^ at 0.1C, 0.2C, 0.5C, 1C, 3C, respectively. The corresponding discharge capacities of LLO are 250.2 mA h g^−1^, 241.2 mA h g^−1^, 223.0 mA h g^−1^, 201 mA h g^−1^, 175.3 mA h g^−1^ at the same current values. As shown in Table S2,† the parameters *a* and *c* of the *R*3̄*m* phase in the Ce-modified samples are larger than that of LLO, which is ascribed to the fact that a portion of Ce^4+^ (*r* = 0.87 Å) has been inserted into the bulk region. In general, the increases in the *c* and *c*/*a* value are considered to enhance the Li^+^ diffusion rate, therefore explaining the improvement of the rate performance of the Ce-modified samples compared with that of LLO. Hence, the appropriate amount of Ce contributes to enhancing the electrochemical performance of cathode materials. On the other hand, the charge-transfer resistance between the electrode and electrolyte is partially reduced owing to the formation of the Li_2_CeO_3_ coating, which is confirmed by the EIS results in the following paragraphs.

The cycling performance test was performed at 0.2C for 100 cycles in the voltage range of 2.0–4.8 V. As presented in Fig. 6b, LLO exhibits an apparent capacity attenuation with a discharge capacity of 182.2 mA h g^−1^ and capacity retention rate of 75.6% after 100 cycles. However, LLO-2 displays an enhanced cycling performance with a capacity of 222.5 mA h g^−1^, and the capacity retention rate is 88.1% after 100 cycles. As shown in Fig. 6b, the capacity retention rate of LLO reaches 80% at the 83rd cycle. However, it is clear that the lifespan of LLO-1, LLO-2, and LLO-3 is 136 cycles, 160 cycles and 142 cycles, respectively, proving that Ce-modification could prolong the life cycle of LLOs (Fig. S6b†). On the other hand, as shown in Fig. 6c, the average discharge voltage degradation of LLO-2 is only 0.32 V after 100 cycles (3.2 mV per cycle). In contrast, LLO delivers an average discharge voltage degradation of about 0.46 V (4.6 mV per cycle). Moreover, LLO-2 exhibits an improved energy retention rate of 79.8% (from 887.4 to 708.4 W h kg^−1^) after 100 cycles, while the energy retention rate of LLO is only 65.1% (from 830.2 to 540.9 W h kg^−1^) after 100 cycles (Fig. 6d). One reason for the enhancement of the cyclic stability is the suppressed interfacial reaction resulting from the protective Li_2_CeO_3_ coating. On the other hand, the subdued transition metals (TMs) migration and Li^+^ irreversible dissolution owing to the suppression of O_2_ release may contribute to restraining the phase transformation from the layered structure to the spinel or rock-salt phase.^47^

To distinguish the impact of bulk doping and surface coating, LLO-4 was prepared by coating Ce on the LLO sample. Fig. S7a† displays the SEM image of LLO-4, in which the morphology of the primary particle is almost similar to that of LLO-2. As listed in Table S2,† the cell parameter *c* of LLO-4 is close to that of LLO, suggesting that almost no Ce has been diffused into the lattice of the bulk (Fig. S7b†). As expected, the discharge capacity of LLO-4 is 255.5 mA h g^−1^ with an initial coulombic efficiency of 86.8% (Fig. S7c†). Compared with LLO-2, the capacity of LLO-4 is 8 mA h g^−1^ lower than that of LLO-2, which may be because almost all of Ce transfers to Li_2_CeO_3_ or CeO_2_ and no Ce diffuses into the bulk in LLO-4, in line with the XRD result. The above results prove that the bulk doping of Ce contributes to enlarging the lattice, which is beneficial for increasing the Li^+^ diffusion rate. As shown in Fig. S7d and e,† the capacity retention rate of LLO-4 is 85.8% and the corresponding average discharge voltage attenuation is 0.38 V (3.8 mV per cycle) after 100 cycles. Moreover, LLO-4 displays an energy retention rate of 76.5% (from 871.8 to 666.7 W h kg^−1^) after 100 cycles (Fig. S7f†). Therefore, the surface coating of Li_2_CeO_3_ is conducive to protecting the electrode from erosion by the electrolyte and suppressing the side reaction, hence enhancing the cycling performance. Meanwhile, the doping of Ce may contribute to enhancing the stability of the structure, when comparing LLO-2 with LLO-4.

**Fig. 7:**
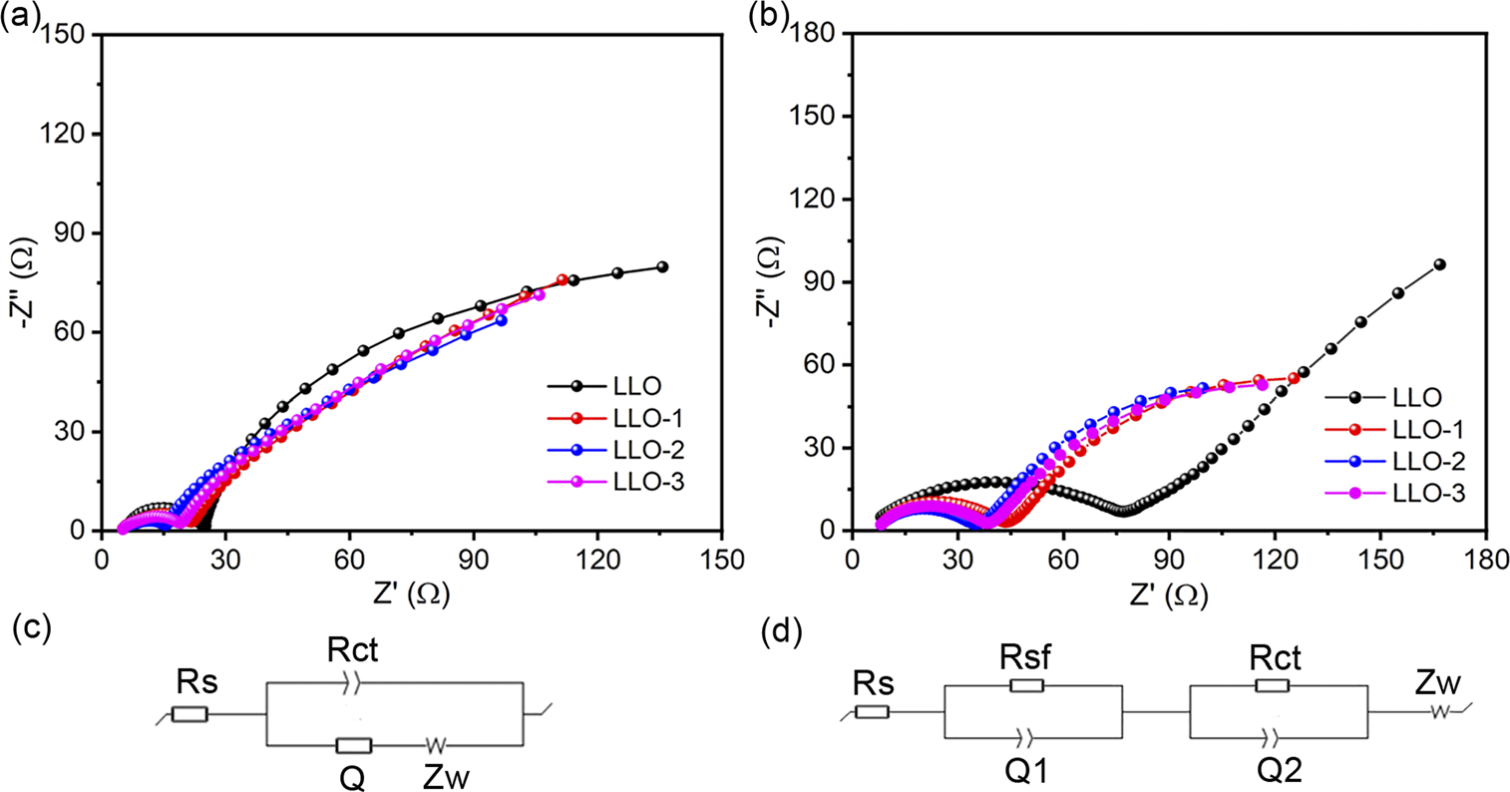
(a) EIS spectra of LLO, LLO-1, LLO-2 and LLO-3 before cycling and (c) the corresponding equivalent circuit, (b) EIS spectra of LLO, LLO-1, LLO-2 and LLO-3 after 100 cycles and (d) the corresponding equivalent circuit.

To further identify the rationale for the improved electrochemical performance of Ce-modified materials, EIS measurement was performed at 4.8 V for the samples before and after 100 cycles at room temperature, as shown in Fig. 7. In general, the high-frequency region is assigned to the ohmic resistance resulting from the resistance of the electrolyte, which is the *R*_s_ in the equivalent circuit. The *R*_sf_ in the equivalent circuit represents the conduction impedance of Li^+^ passing the surface electrolyte film (SEI) on the surface of the electrode. *R*_ct_ is the charge-transfer resistance and *Z*_w_ is the Warburg impedance. The fitting parameters are listed in Table S3.† It can be seen that the impedance of the Ce-modified samples is smaller than that of the pristine one, particularly for LLO-2. After 100 cycles, LLO-2 still exhibits the least *R*_ct_ value compared with LLO, which suggests that LLO-2 has a much more stable interface. As shown in Table S3,† the *R*_ct_ of the pristine sample increases more than that of the modified samples, which is due to the severe phase transition and structure degradation. This result demonstrates that the Li_2_CeO_3_ coating layer can effectively inhibit the phase transformation and stabilize the interface between the electrode and the electrolyte. In short, the Li_2_CeO_3_ coating layer can significant enhance the stability of the structure and improve the electrochemical performance.

**Fig. 8:**
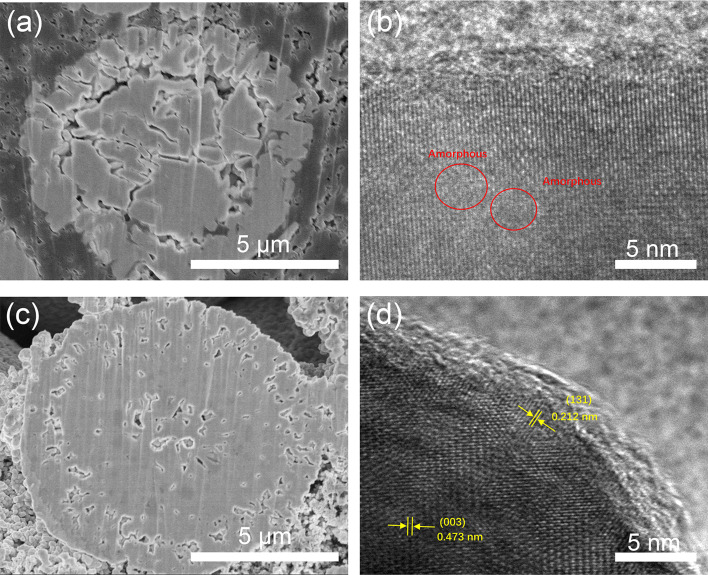
(a) SEM image of cross-section of LLO and (b) HRTEM image of LLO after 100 cycles, (c) SEM of cross-section of LLO-2 and (d) HRTEM image of LLO-2 after 100 cycles.

In order to further investigate the effect of Ce coating, the XRD patterns of LLO and LLO-2 after the cycles are provided. As shown in Fig. S8,† the (003) diffraction peak of LLO is much weaker than that of LLO-2, indicating the less crystallinity of LLO compared with LLO-2 after 100 cycles, which may result from the migration and dissolution of transition metals and lattice oxygen loss. Moreover, SEM and HRTEM analyses of LLO and LLO-2 after 100 cycles were performed. As displayed in Fig. 8a and b, the spherical shape of LLO had collapsed completely and the partial layered structure was transformed into amorphous phase. On the contrary, almost no obvious crack was observed in the bulk of LLO-2 (Fig. 8c). Moreover, the Li_2_CeO_3_ coating still exists on the surface of the LLO-2 sample, in which the amorphous residue on the Li_2_CeO_3_ coating may be PVDF or carbon black (Fig. 8d). Hence, the above results demonstrate that the Li_2_CeO_3_ coating layer could inhibit the phase transformation, which usually worsens the cycling performance of Li-rich materials. These SEM and HRTEM results are in accordance with the above XRD analysis, which prove that Li_2_CeO_3_ coating on the Li-rich materials contributes to improving the capacity and retarding the voltage decay.

Meanwhile, XPS of LLO and LLO-2 electrodes after cycling was carried out to further analyze the structural changes. As displayed in Fig. S9a,† the survey spectrum of LLO is much weaker than that of LLO-2. Also, the Ni 2p and Mn 2p peaks of LLO almost disappear entirely while the corresponding peaks of LLO-2 still exist, which demonstrates that the dissolution of Ni and Mn in LLO-2 is alleviated in comparison with that in LLO (Fig. S9b and c†). As shown in Fig. S9d,† the O 1s spectra are split into three peaks at 529.5 eV, 531.0 eV and 533.0 eV, which are assigned to lattice oxygen, oxygen vacancy and chemisorbed oxygen, respectively. It is obvious that the peak at 529.5 eV for LLO-2 is stronger than that of LLO, demonstrating less lattice oxygen loss in LLO-2. Moreover, LLO-2 still has a certain amount of oxygen vacancies after the cycles compared with LLO. Also, the weaker chemisorbed oxygen peak of LLO-2 proves that electrolyte decomposition has been suppressed significantly, which can account for the enhanced cycle stability of LLO-2 to a degree.

## Conclusion

In summary, the effect of Ce-modification on the structure, surface chemistry and electrochemical performances of Li-rich Mn-based layered oxides materials were investigated. TEM, XRD and XPS analyses prove that Ce diffuses into the lattice successfully and the LiCe_2_O_3_ coating is formed on the surface of LLOs. The cell test shows that Ce doping could enhance the ICE and cycle properties of LLOs. More importantly, the Li_2_CeO_3_ coating with oxygen vacancies on the surface of the electrode could protect the electrode from erosion by the electrolyte, suppress the side reaction, and reduce oxygen release in the bulk, therefore improving the electrochemical performances. As a consequence, the as-prepared LLO-2 exhibits an enhanced rate performance (186.6 mA h g^−1^ at 3C) and better cycling stability (88.1% capacity retention rate after 100 cycles at 0.2C). Therefore, this Ce-modification strategy could be a beneficial methodology to overcome the intrinsic drawbacks of Li-rich Mn-based layered oxides.

## Data availability

The data supporting this article have been included as part of the ESI.†

## Author contributions

Shaofeng Liu: conceptualization, experimental investigation, writing original draft. Haifeng Yue: SEM and TEM measurements. Yan Mo: conceptualization, experimental investigation, writing original draft. Liang Luo: XRD measurement and Rietveld refinement. Xiaozhen Wu: review & editing. Shunyi Yang: review & editing. Youyuan Huang: supervision, review & editing. Guohui Yuan: supervision, financing, review & editing.

## Conflicts of interest

The author declare that they have no known competing financial interests or personal relationships that could have appeared to influence the work reported in this paper.

## Supplementary Material

RA-014-D4RA03660C-s001
