# Supplementary material for: Retarding the capacity fading and voltage decay of Li-rich Mn-based cathode materials via a compatible layer coating for high-performance lithium-ion batteries
Source: RSC Adv. 2024 Aug 19;14(36):26142–51. doi: 10.1039/d4ra03660c (PMC11331581; doi:10.1039/d4ra03660c)
Supplement: RA-014-D4RA03660C-s001 [file RA-014-D4RA03660C-s001.pdf]

Supplementary Information (SI) for RSC Advances.
This journal is © The Royal Society of Chemistry 2024

Supporting Information

Retarding the capacity fading and voltage decay of Li-rich Mn-based cathode material via compatible layer coating for high performance lithium-ions batteries

Shaofeng Liu,‡[a,b] Haifeng Yue,‡[a,b] Yan Mo,[a,b] Liang Luo,[b] Xiaozhen Wu,[b] Shunyi Yang,[b] Youyuan Huang*[b] and Guohui Yuan*[a]

[a] *School of Chemistry and Chemical Engineering, Harbin Institute of Technology, Harbin 150001, People's Republic of China.*

[b]*Shenzhen BTR nanotechnology Co., Ltd., Shenzhen 518106, People's Republic of China.*

**E-mail: ygh@hit.edu.cn, huangyouyuan@btrchina.com*

‡ These authors contributed equally.

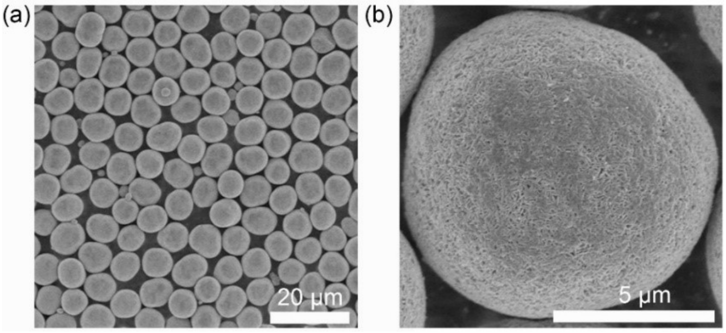

Fig. S1 SEM image of as-prepared precursor.

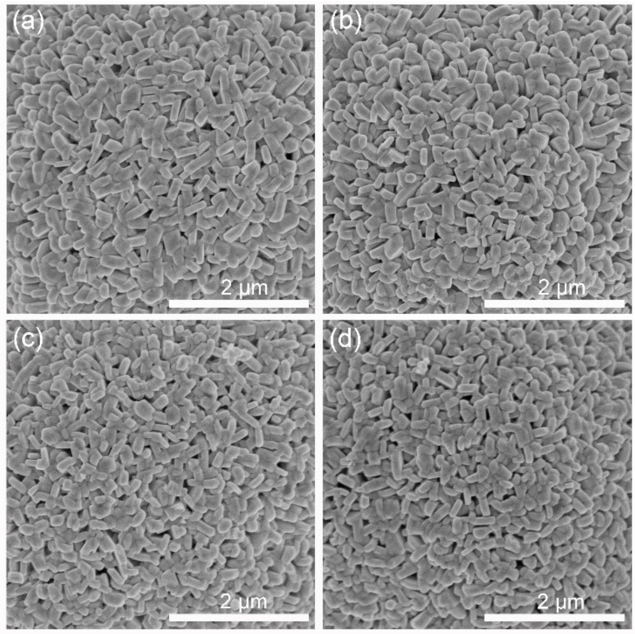

Fig. S2 Enlarged SEM images of (a) LLO, (b) LLO-1, (c) LLO-2 and (d) LLO-3.

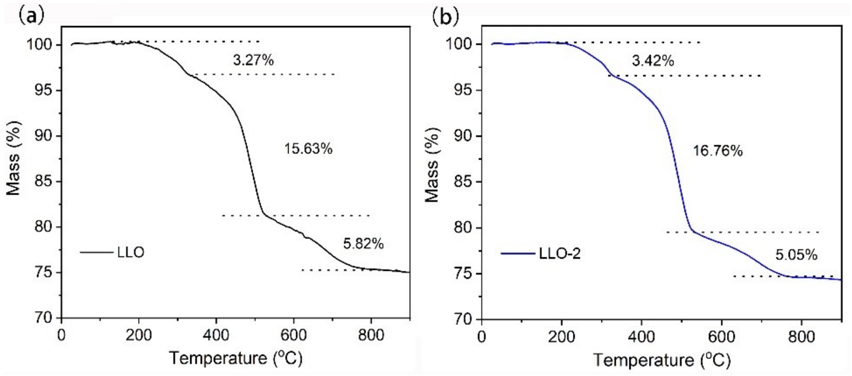

Fig. S3 TG analysis of (a) LLO and (b) LLO-2.

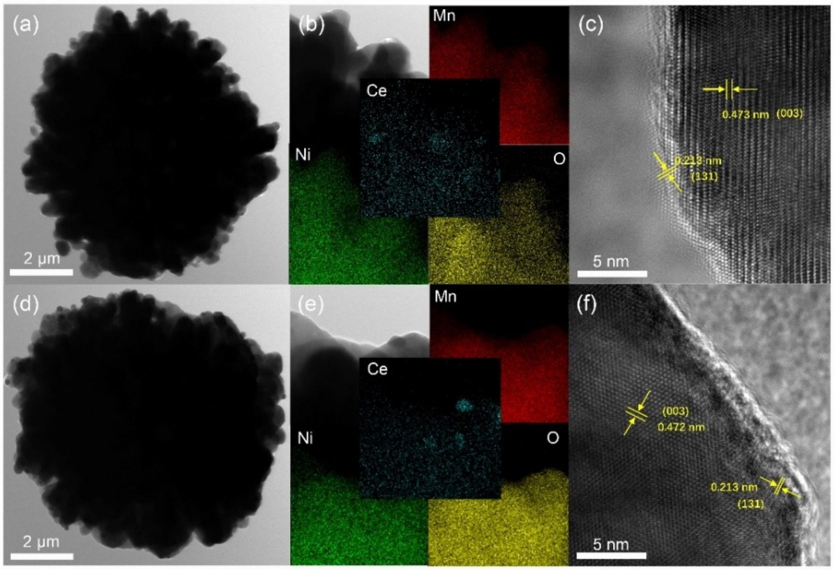

Fig. S4 (a) TEM image, (b) EDS elemental mapping and (c) HRTEM image of LLO-1, (d) TEM image, (e) EDS elemental mapping and (f) HRTEM image of LLO-3.

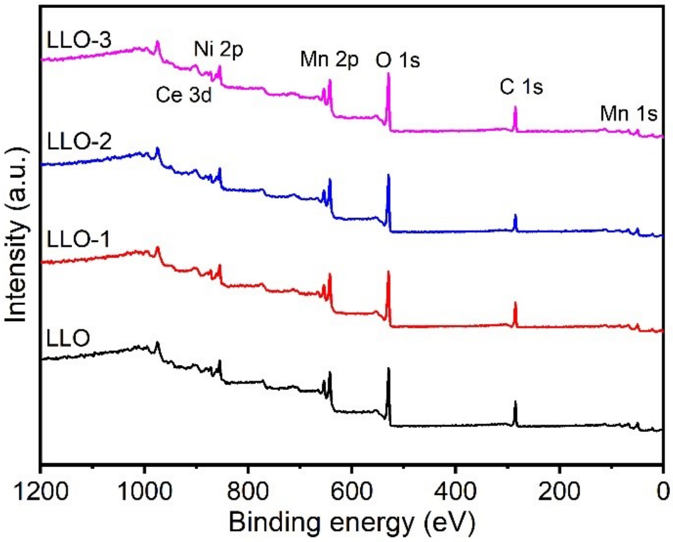

Fig. S5 XPS survey spectra of pristine and Ce-modified samples.

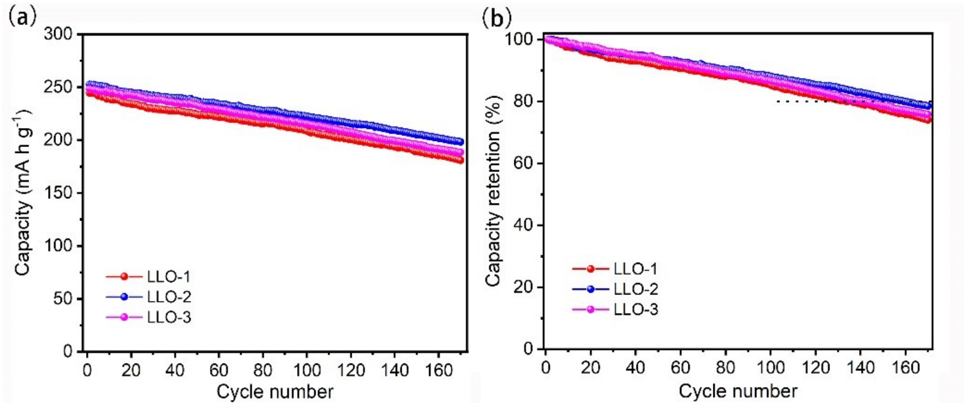

Fig. S6 (a) Discharge capacity and (b) corresponding capacity retention vs. cycle number for LLO-1, LLO-2 and LLO-3 at 0.2C between 2.0 and 4.8 V.

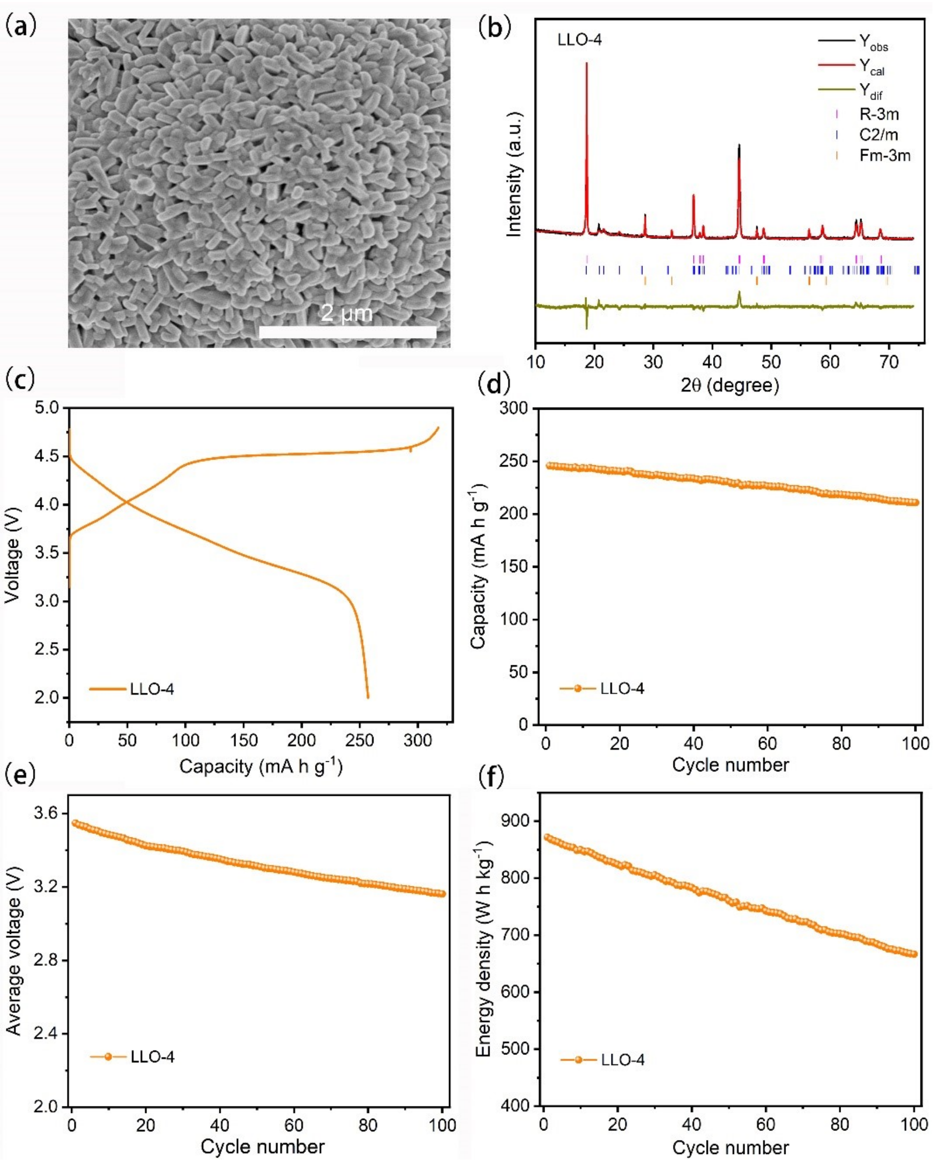

Fig. S7 (a) SEM image and (b) Riveted refined XRD profiles of LLO-4, (c) initial charge-discharge curve profiles, (d) capacity retention, (e) median voltage decay, (f) energy density retention of LLO-4 at 0.2C between 2.0 and 4.8 V.

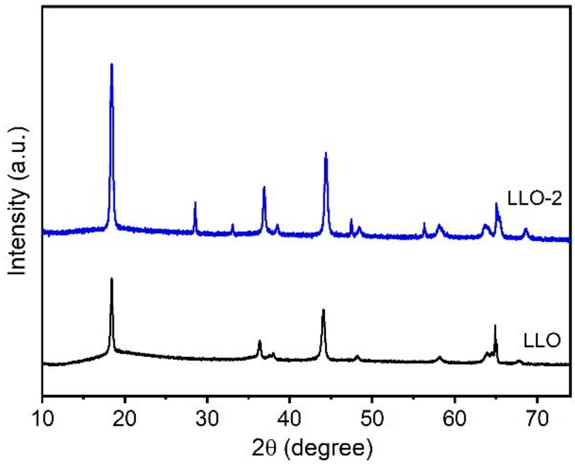

Fig. S8 XRD patterns of LLO and LLO-2 after cycling.

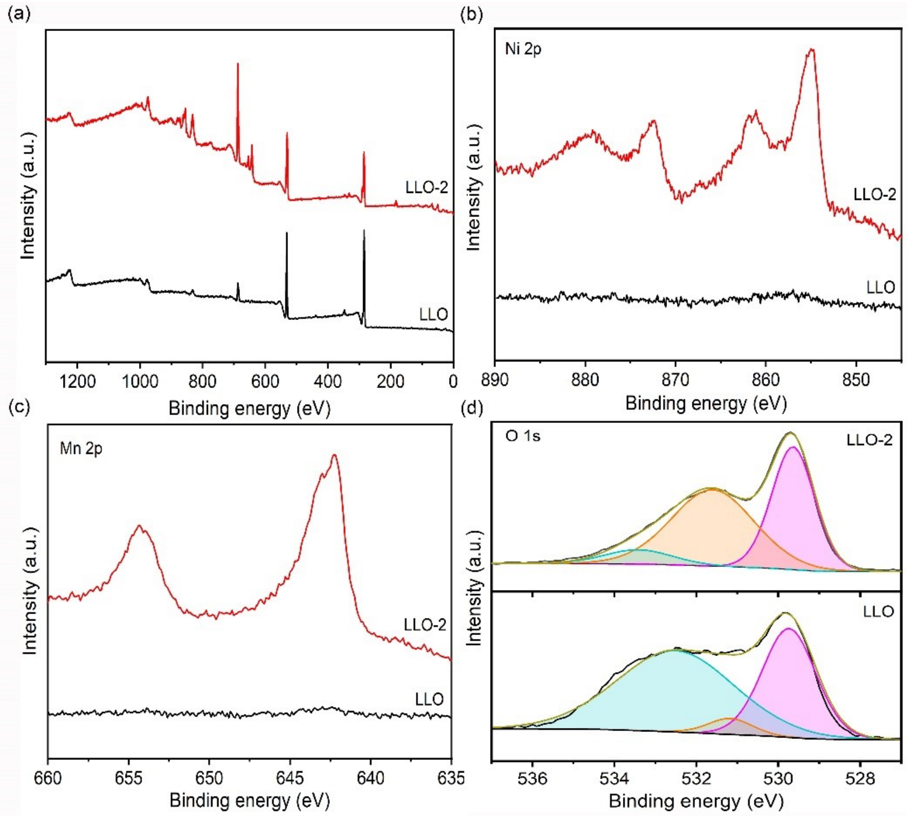

Fig. S9 XPS spectra of (a) full spectra, (b) Ni 2p, (c) Mn 2p, (d) O 1s for LLO and LLO-2 after 100 cycles at 0.2C.

Table S1. ICP-OES results for as-prepared materials

Sample	Li (%)	Ni (%)	Mn (%)	Ce (%)
LLO	9.79	13.42	38.81	0
LLO-1	9.79	13.51	38.54	2.92
LLO-2	9.79	13.37	38.65	4.98
LLO-3	9.79	13.47	38.92	6.89

Table S2. Results of XRD Rietveld refinement

Sample			LLO	LLO-1	LLO-2	LLO-3	LLO-4
	R-3m	a	2.8599	2.8615	2.8626	2.8609	2.8602
		c	14.2514	14.2725	14.2753	14.2556	14.2519
Lattice		c/a	4.983	4.988	4.987	4.983	4.983
parameters	C2/m	a	4.9564	4.9466	4.9548	4.9352	4.9674
		b	8.5788	8.6013	8.5785	8.5886	8.5392
		c	5.0372	5.0813	5.0378	5.0829	5.0431
	Fm-3m	a		5.4149	5.4073	5.4146	5.4078
Weight	R-3m		75.33	72.99	74.68	71.47	61.36
Fraction	C2/m		24.67	25.84	22.34	23.63	36.29
(%)	Fm-3m			1.17	2.98	4.90	2.36
Reliability	Rwp		5.20	4.93	5.96	4.97	2.71
Factor	Rp		3.32	3.30	4.28	3.32	4.01

Table S3. Fitted parameter of EIS spectra of prepared samples for before and after 100 cycles

	Samples	LLO	LLO-1	LLO-2	LLO-3
1st	R_s/Ω	6.65	5.51	4.81	5.09
	R_{ct}/Ω	14.92	14.36	8.49	12.05
100th	R_s/Ω	4.94	6.91	6.26	5.69
	R_{sf}/Ω	76.25	35.40	28.92	32.63
	R_{ct}/Ω	267.8	165.9	157.2	179.1
